# Taxonomic composition and seasonal dynamics of the air microbiome in West Siberia

**DOI:** 10.1038/s41598-020-78604-8

**Published:** 2020-12-09

**Authors:** Elena S. Gusareva, Nicolas P. E. Gaultier, Balakrishnan N. V. Premkrishnan, Carmon Kee, Serene Boon Yuean Lim, Cassie E. Heinle, Rikky W. Purbojati, Ang Poh Nee, Sachin R. Lohar, Koh Yanqing, Vladimir N. Kharkov, Daniela I. Drautz-Moses, Vadim A. Stepanov, Stephan C. Schuster

**Affiliations:** 1 Singapore Centre for Environmental Life Sciences Engineering (SCELSE), Nanyang Technological University, 60 Nanyang Drive, Singapore, 637551 Singapore; 2 Research Institute of Medical Genetics, Tomsk National Research Medical Centre, Russian Academy of Sciences, Tomsk, Russian Federation 634050

**Keywords:** Biodiversity, Ecosystem ecology, Microbial ecology, Population dynamics

## Abstract

Here, we describe taxonomical composition, as well as seasonal and diel dynamics of airborne microbial communities in West Siberia. A total of 78 airborne biomass samples from 39 time intervals were analysed, within a temperature range of 48 °C (26 °C to − 22 °C). We observed a 5–170-fold decrease in DNA yield extracted from the airborne biomass in winter compared to summer, nevertheless, yielding sufficient material for metagenomic analysis. The airborne microbial communities included Actinobacteria and Proteobacteria, Ascomycota and Basidiomycota fungi as major components, as well as some Streptophyta plants. In summer, bacterial and fungal plant pathogens, and wood-rotting saprophytes were predominant. In winter, Ascomycota moulds and cold-related or stress environment bacterial species were enriched, while the fraction of wood-rotting and mushroom-forming Basidiomycota fungi was largely reduced. As recently reported for the tropical climate, the airborne microbial communities performed a diel cycle in summer, however, in winter diel dynamics were not observed.

## Introduction

The field of bioaerosol research studies the taxonomy and community composition of airborne microbial organisms, also referred to as air microbiome. A recent series of technological and analytical advancements include high-volumetric air samplers, an ultra-low biomass processing pipeline, low-input DNA sequencing libraries, as well as high-throughput sequencing technologies. Applied in unison, these methods have enabled comprehensive and meaningful characterization of the airborne microbial organismal dynamics found in the near-surface atmosphere^1^. Previous studies investigating bioaerosols using amplicon sequencing predominantly focussed on the bacterial fraction of the air microbiome, while fungal and plant pollen fractions frequently remained understudied^2–9^. Recent reports show the effect of airborne microbial communities on human health, driven by respiratory pathogens^10–12^ and agents that trigger allergies^13–15^. Airborne microbial organisms also impact agricultural productivity, as bacterial and fungal species distributed by air movement act as plant blights^16^. Furthermore, atmospheric processes, such as cloud condensation and ice nucleation events were shown to depend on airborne microbial particles^17^. Therefore, understanding the dynamics of microbial organisms in air is crucial for insights into the atmosphere as an ecosystem, but also will inform on respiratory health aspects and human wellbeing.

In seasonal climate settings, assemblages of airborne microorganisms are dynamic and vary across seasons^3,18,19^ and from day-to-day^4^. Compositions of the airborne communities may also vary between locations^20^, however, meteorological factors appear to have a stronger effect on microorganisms than other factors of the local environment^4, 9^. Recently, we have shown that in a tropical climate, airborne microbial communities are stable across days, weeks and months for samples taken at the same time of the day, while significant fluctuations in microbiome composition occur across different time points within a day. These diel air microbiome oscillations are correlated with atmospheric temperature, relative humidity and CO_2_ daily profiles^1^. In seasonal climate settings, however, seasonal and diel changes of airborne communities are currently understudied.

We sampled airborne biomass in West Siberia (Yurga, 55.711 N, 84.937 E), an extensive geographical region of Russia with a continental climate. The recorded average temperatures range from 6 to 24 °C in summer (June–August), and from − 21 to − 6 °C in winter (November–March). In most years, snow accumulation occurs from October/November until March/April. Meteorological characteristics (temperature, relative humidity, and wind direction) during the time-series experiments (Fig. S1, S2) represent typical summer and winter settings of the region. In this report, we provide a metagenomic airborne community analysis of three time-series surveys of the near-surface atmosphere in two seasonal settings. This culture-independent study of airborne microorganisms identifies the bacterial, fungal and plant genetic material with a high taxonomic and temporal resolution in a single sampling set-up. Hence, the relative abundance of the entire airborne community can be represented on the same scale.

The analysis presented here demonstrates the impact of seasonal and diel changes in the community composition of airborne microorganisms and reveals temperature as the key driver for the observed ecosystem dynamics.

## Results

We conducted three time-series surveys collecting airborne biomass during 2 h time intervals 3–4 times per day for 2 to 5 days in summer and in winter 2017 and 2018 (see Methods). In total, 78 airborne biomass samples from 39 time-points were collected (Table S1). The amount of biomass collected by our air sampling approach was assessed by quantification of extracted DNA. The metagenomic analysis of the extracted DNA was performed using the RAPSearch aligner^21,22^ and MEGAN^23,24^ as a taxonomic classifier on Illumina whole genome shotgun sequencing data.

**Figure 1:**
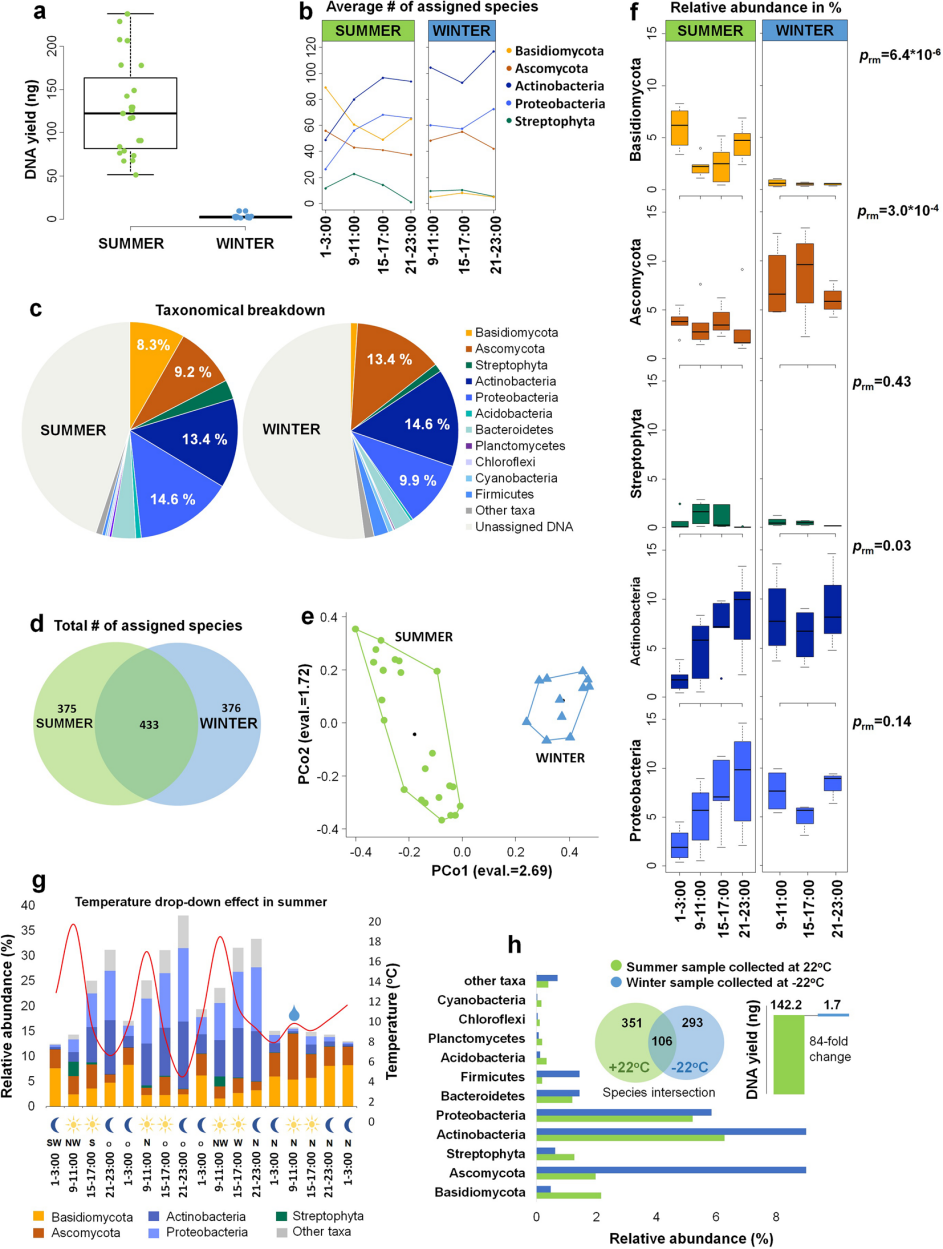
Seasonal dynamics of the airborne microbial communities. (**a**) Total DNA yields (in ng) isolated from the collected airborne biomass samples during the summer and winter time-series. (**b**) Richness of the summer and winter microbial communities with respect to the five most abundant taxa (Basidiomycota, Ascomycota, Actinobacteria, Proteobacteria, and Streptophyta). Airborne biomass samples were collected in different time intervals as indicated on the X axis. (**c**) Structure of the summer and winter microbial communities in maximal relative proportions of taxa. (**d**) Species intersection between summer and winter time-series. Values indicate the total number of identified species during summer and winter time-series. (**e**) Bray–Curtis dissimilarity distances between summer and winter samples plotted in the first two principal coordinates (PCo1 and PCo2). (**f**) Diel dynamics of the five most abundant airborne microbial taxa in summer and winter. *P*-values indicate significance of the differences in relative abundances between the 5 taxonomical groups in summer and winter time-series (regression modelling analysis). (**g**) Temperature drop-down effect in summer. Four consecutive days in summer 2018 time series are represented. Relative abundances (in %) of the five most abundant taxa are plotted. Red curve represents atmospheric temperature profile during the days of the experiment. Time scale is indicated at the bottom of the figure. Day and night samples denoted by sun and moon symbols. A rain event is indicated by the water drop-shaped symbol. Wind direction is indicated as follows: S—south, SW—southwest, NW—northwest, N—north, o—calm period. (**h**) Comparison of the summer and winter samples collected at + 22 °C and − 22 °C, respectively. Histogram represents relative abundances (in %) of taxa in both samples. Venn-diagram represents species intersection between the two samples. Absolute biomass load is presented via DNA yield (in ng).

Large differences in biomass quantity were observed between summer and winter air samples (Fig. 1a). Total DNA yield extracted from the airborne biomass samples in summer ranged from 48.0 to 237.6 ng, while in winter only 1.4 to 9.6 ng of DNA could be obtained. Our sampling methods were performed in winter at temperatures as low as − 22 °C. Despite up to 170-fold differences between summer and winter samples (39.3-fold on average, Fig. 1a), sampling intervals of 2 h yielded sufficient biomass for DNA metagenomic sequencing and deep metagenomic analysis, resulting in species level taxonomic identification. Independent of the season, a 3–5-fold fluctuation in the extracted DNA yield was observed within 24 h (Fig. S3, upper panel). Regardless of season, the majority of sequencing reads were assigned to bacterial taxa (~ 2–38%), followed by eukaryotes (~ 4–16%, mostly fungal), and < 1% to archaea, with traces of phages (Fig. S3, middle panel). Despite temperature differences of 48 °C across our sampling series, a consistently high species diversity was noted, as detected by our in-depth taxonomical analysis (Fig. 1b,c). Number of assignable taxa per 2 h sampling interval ranged from 200 to 374 (11.8–42.2% of all reads) (Fig. S3, middle and lower panels), using a cut-off value of 25 reads per taxon (see Methods).

The prevailing microbial taxa were Proteobacteria and Actinobacteria, Ascomycota and Basidiomycota fungi, and Streptophyta plants (Fig. 1c). When intersecting all time-series samples, 433 species represented the core of the microbial communities, both in summer and in winter (Fig. 1d). The majority were bacteria (315 species), followed by fungi (89 species) and species belonging to other taxonomic groups (29 species) (Fig. S4). Despite a substantial fraction of species occurrence was overlapping between summer and winter (Fig. 1d), principle coordinate analysis clearly segregates both groups (Fig. 1e), driven by the Bray–Curtis dissimilarity distances of the most abundant organisms.

### Summer air microbiomes

We detected a high diversity of Ascomycota and Basidiomycota fungi (Fig. S5 and S6) in summer samples, with the majority representing wood rotting saprophytes (*Schizopora paradoxa, Dichomitus squalens, Phanerochaete carnosa, Trametes pubescens, Phlebiopsis gigantea, Fomitopsis pinicola, Glarea lozoyensis, Phlebia centrifuga, Stereum hirsutum,* etc.). Other taxa were plant pathogens (*Botrytis cinerea, Alternaria alternata, Rhizoctonia solani, Epicoccum nigrum, Parastagonospora nodorum, Puccinia striiformis,* etc.), as well as common mushroom fruiting body-forming fungi (*Pleurotus ostreatus, Agaricus bisporus, Hypsizygus marmoreus,* and *Hypholoma sublateritium*) (Table S2). The majority of identifiable bacteria were aerobic mesophilic Proteobacteria and Actinobacteria previously isolated from air, fresh water or wastewater, and plants (Table S2). Several cold-related actinobacterial species were also identified (e.g., *Cnuella takakiae, Arthrobacter *sp.* L77, and Kocuria polaris,* etc.) (Table S2). Plant species that are part of the typical flora of West Siberia, were among the top-ranked airborne taxa (*Helianthus annuus, Vitis vinifera, Morus notabilis*, and *Daucus carota* in summer 2017, and *Aegilops tauschii* in summer 2018; Table S2).

The relative abundance (Fig. 1f) and richness of the bacterial community (Fig. 1b) reached their maximum values in the evening hours (21:00–23:00), and significantly decreased at night (1:00–3:00). The fungal community showed complementary behaviour with the highest relative abundance and richness observed at night (1:00–3:00), while decreasing during day. The day/night airborne microbial community structure was significantly distinct (multivariate linear modelling analysis in the Summer 2018 time-series: *p*_Multivariate_GLM_-value = 0.036). In this regard, certain groups of microbial taxa, mostly Basidiomycota fungi, were significantly more abundant at night (*p*_Multivariate_GLM_-value < 0.05, Fig. S7). We also detected a cross-correlation between the atmospheric temperature daily oscillation profiles and relative abundances of some taxa (Fig. S8). This cross-correlation with temperature was specifically strong for Basidiomycota fungi (*r* = − 0.58) and Streptophyta plants (*r* = 0.82). Moreover, we observed the effects of a rapid drop in temperature on the airborne microbial community dynamics and structure (Fig. 1g). In this regard, in the last day of the Summer 2018 time-series, northern winds caused a drop of the recorded temperature to 10 °C and accompanying light rain, resulting in a shifted air microbiome composition that was prominently decreased in bacterial taxa, with elevated abundances of Ascomycota and Basidiomycota fungi (Fig. 1g).

### Winter air microbiomes

Winter microbial communities were enriched in cold- or stress-tolerant environmental bacterial and fungal species (*Wallemia mellicola, Rachicladosporium antarcticum, Hymenobacter sp. PAMC 26,554, Arthrobacter sp. L77, Kocuria polaris, Hymenobacter roseosalivarius, Thermoactinomyces sp. CDF*, etc.) (Table S2). Also, *Aspergillus* moulds were more prolific in colder temperature settings, in contrast to wood-rotting and mushroom-forming Basidiomycota fungi (Fig. 1c,f, Table S2, Fig. S9). Notably, the Gram-positive bacterium *Saccharopolyspora rectivirgula* was among the most abundant airborne bacteria in winter settings (Table S2, Fig. S9). This bacterium is a causative agent of farmer's lung disease, a type of hypersensitivity pneumonitis in immunocompromised patients^25^.

Despite the lack of perennial plant materials during winter in Siberia, we observed airborne biomass of cereal plants (Poaceae) (e.g., *Aegilops tauschii*, *Triticum Urartu*, *Oryza sativa*, *Hordeum vulgare*) (Table S2). This is likely due to the fact that the stalks of cereals can rise above snow level, thus allowing the spikes to continuously spread their biological material through the air, even after the growth season has ended.

Throughout the winter time-series, no diel variation was observed for any of the five taxonomic groups (Ascomycota, Basidiomycota, Streptophyta, Actinobacteria and Proteobacteria) (Fig. 1f).

## Discussion

Sampling of airborne biomass is technically challenging due to low amounts of collectable material of a given size (0.5–10 µm). Therefore, research into the dynamics of airborne microbial communities is often restricted to long sampling periods. In this study, we established a sampling and analysis regime that enabled the successful sequencing of ultra-low biomass samples, collected during 2 h time-intervals and at temperatures that were as low as − 22 °C during the Siberian winter (Fig. 1h).

Our study revealed striking seasonal differences with up to 170-fold higher DNA yield extracted from the collected biomass in summer. In winter, due to low temperatures and snow cover, the potential sources and sinks of the near surface atmosphere harbour distinctly altered airborne microbial communities. This is largely due to the inaccessibility of perennial plant material and is documented by the strong reductions in Streptophyta and Basidiomycota (Fig. 1c,f), which include wood-rotting fungi and mushroom fruiting bodies. In contrast, the fraction of the mould-forming Ascomycota is strongly increased in winter, while in summer, Ascomycota are less abundant and represented mostly by plant pathogens (Fig. 1c,h, Table S2). For the bacterial domain, the most striking seasonal change is noted for richness and relative abundance of Firmicutes, with relatively lower differences observed for Proteobacteria (Fig. 1h, Fig. S10, Table S3). Despite this observation, the overall richness of the winter microbial community was comparable to summer communities (Fig. 1b).

The temporal resolution of our sampling approach enabled the diel dynamics of the air microbiome to be documented for continental climates. In summer, when the day/night atmospheric temperature differences become more prominent, members of the microbial community follow a diel cycle (Fig. 1f, summer), similar to the one previously reported for tropical settings^1^. These diel oscillations are the most pronounced in Actinobacteria and Proteobacteria, but also in Basidiomycota fungi and Streptophyta plants (Fig. 1f). Changes in temperature, and the interconnected relative humidity are likely the most important drivers of these diel oscillations, while the duration of daylight appears less important. Interruptions in the diel temperature cycle by a sudden decrease of the atmospheric temperature instantly affected community composition (Fig. 1g). In winter, when the temperature and relative humidity differences are low, the dynamics of the diel air microbiome is diminished (Fig. 1f, winter). Further factors impacting microbial community compositions are emissions as such reported during seasonal air pollution events in Siberia^26^, which potentially may impact the summer and winter dynamics of the local air microbiome.

Our study comprehensively characterizes airborne microbial communities in West Siberia and provides a direct comparison between bacteria, fungi, as well as other organisms that release biological matter into the environment. In contrast to earlier studies of bioaerosols in West Siberia^18,19,27,28^, our approach is cultivation free and not based on nucleic acid amplification. Therefore, direct comparison of the microbial community structures between this study and previously published ones is challenging. Nevertheless, the observation of the seasonal dynamics of airborne bacteria and fungi in West Siberia is concordant with previous studies^18,19,27^. Furthermore, the cultivation-based identification of the airborne bacterial^28^ and fungal taxa^19^ confirms on higher taxonomic level the results from our study, which in contrast allows for a species level analysis (Table S2). The observation of the diel cycle occuring also in the West Siberian air ecosystem, however, could only be made using the technological advances first demonstrated in the Gusareva et al.^1^ publication and in this study, as the previous methods did not allow for a sufficient temporal resolution.

### Conclusion

The airborne biomass consisting of microbial communities was studied in West Siberia by metagenomic analysis for the first time. A large difference in biomass, assessed by DNA analysis, existed for the summer and winter seasons. Despite an up to 170-fold reduction in microbial and plant material, the deployed sampling protocol yielded quality DNA libraries with high complexity. In summer, when the atmospheric temperature and relative humidity fluctuate in a day/night pattern, the airborne microbial communities followed a diel cycle, as previously described for the tropical climate. This diel fluctuation was absent during consistently cold temperatures encountered in the winter months.

## Methods

### Time-series sampling

Air samples were collected in Yurga (55.711 N, 84.937 E), where the average temperatures range from 6 to 24 °C in summer (June–August), and from − 21 to − 6 °C in winter (November–March) (the open source service https://weatherspark.com/). Meteorological characteristics (temperature, relative humidity, and wind direction) during the time series are represented in Fig. S1 and S2. Specifically, air samplers were positioned at an open-air balcony (~ 4 m above the ground level under a concrete canopy) of a five-storey residential setting. Samples were collected in duplicates (i.e., two technical replicates) with two high flowrate and filter-based air samplers (SASS3100, Research International, USA). The first set of samples were collected during three time periods (1:00–3:00, 9:00–11:00, and 15:00–17:00) on 26 and 28 July 2017; the second set was also collected during three time periods (9:00–11:00, 15:00–17:00, and 21:00–23:00) within consecutive days from 2 to 5 December 2017, the third set was collected during four time periods (1:00–3:00, 9:00–11:00, 15:00–17:00, and 21:00–23:00) within consecutive days from 27 August to 2 September, 2018. In total, 78 samples in 39 time intervals were collected and used for preparation of 62 sequencing libraries (Table S1).

High volumetric, filter-based air samplers (SASS3100, Research International, USA) were used in this study, with SASS bioaerosol electret filters (6 cm diameter, expected 50% efficiency for 0.5 µm particle size, Research International, USA) as the filter medium. Sampling was performed at 300 L/min air flowrate for 2 h. After sampling, the SASS filters were stored at − 20 °C. During transport from Siberia to Singapore, the samples were hand-carried with cooling.

### Sample blanks

In each sampling set, blanks were also collected as controls. The blanks consisted of 12 filter blank samples (FB) and three reagent blank samples (RB). The filter blank samples were collected by installing a new filter on the air sampler at the sampling location for about 5 s. The filter was then collected and analysed with the same protocol as the time-series samples. Reagent blank samples involved extractions performed with extraction reagents without any filter.

Details on metagenomic analysis for blanks are provided in the Supplementary section (Fig. S11).

### DNA extraction

Technical replicates were isolated separately. For processing, the SASS filter was first transferred into a sterile 5 mL tube. Phosphate buffered saline (pH 7.2) with 0.1% (v/v) Triton X-100 (2 mL, PBS-T) was added to the 5 mL tube as the wash buffer. Using tweezers, the SASS filter in the tube was moved up and down a few times to let the PBS-T penetrate the filter. The tube was then sonicated for 1 min in a sonication bath without heating to dislodge the biomass from the filter. After sonication, the filter was squeezed with tweezers and the PBS-T with suspended particles was transferred into a sterile 50 mL conical tube to complete the first washing step. This washing step was repeated three times for each filter sample, using fresh 2 mL PBS-T for each repeat. At the end of the second and third repeats, the filter was transferred into the barrel of a 10 mL syringe, placed in the same 50 mL conical tube containing the wash liquid. The 50 mL tube with the syringe and SASS filter was then centrifuged at 5000×*g* for 2 min to remove any leftover PBS-T. The expected total recovered supernatant volume from the three washes for each sample was 6 mL, which contained the captured airborne particles.

Upon completion of the wash steps, the supernatant was subsequently filtered through a 0.02 µm Anodisc filter (Whatman, UK) using a vacuum manifold (DHI, Denmark). The Anodisc was finally transferred into a 5 mL bead tube provided in the DNeasy PowerWater Kit (Qiagen, Germany) for DNA extraction.

DNA extraction from the Anodisc was mostly performed following the standard protocol of the DNeasy Power Water Kit with the following modifications to increase DNA yield. Briefly, 0.1 mg/mL (final) Proteinase K was added to the lysis buffer (solution PW1) prior to the initial 55 °C incubation. The initial incubation time at 55 °C was also prolonged from the recommended 10 min to overnight incubation. After initial incubation, the sample tubes were vortexed for 3 min and subsequently placed into an ultrasonic bath (Elmasonic, USA) for sonication at 65 °C for 30 min^29^, followed by another 5 min vortex. The remaining extraction steps were completed as instructed in the manufacturer’s protocol.

In the first and second time series (SUMMER 2017 and WINTER 2017), the DNA isolated from the technical replicates was pooled to provide sufficient material for sequencing.

### Metagenomic sequencing

For the metagenomic sequencing and NGS data processing, we used standardised procedures and pipelines described in detail elsewhere^1^. Extracted air DNA samples were quantitated on a Qubit 2.0 fluorometer, using the Qubit dsDNA HS (High Sensitivity) Assay Kit (Invitrogen). Immediately prior to library preparation, sample quantitation was repeated on a Promega QuantiFluor fluorometer, using Invitrogen’s Picogreen assay.

Next-generation sequencing libraries were prepared with Swift Biosciences’ Accel-NGS 2S Plus DNA Library Kit, following the instructions provided in the kit. With the exception of samples that had a concentration of < 0.25 ng/µL, the starting amount of DNA for library preparation was normalized to 5 ng. DNA shearing was performed on a Covaris E220 focused-ultrasonicator with the following settings: peak power: 175, duty factor: 5.0, cycles/burst: 200, run time: 90 s. All libraries were dual-barcoded, using Swift Biosciences’ 2S Combinatorial Dual Indexing Kit. For PCR amplification, which selectively enriches for library fragments that have adapters ligated on both ends, the PCR cycles were normalized to eight for all libraries with a starting amount of 4–5 ng of DNA. For samples with less than 4 ng of DNA, amplification cycles were adjusted as follows: 3.0–3.9 ng: 9 cycles, 2.0–2.9 ng: 11 cycles, 1.0–1.9 ng: 13 cycles, < 1 ng: 15 cycles. Size-selection was omitted for all libraries.

Library quantitation was performed using Invitrogen’s Picogreen assay and the average library size was determined by running the libraries on a Bioanalyzer DNA 7500 chip (Agilent). Library concentrations were normalized to 4 nM and the concentration was validated by qPCR on a ViiA-7 real-time thermocycler (Applied Biosystems), using Kapa Biosystem’s Library Quantification Kit for Illumina Platforms. Libraries were then pooled at equal volumes and sequenced on Illumina HiSeq2500 rapid runs at a final concentration of 10–16 pM and a read-length of 251 bp paired-end (Illumina V2 Rapid sequencing reagents).

### High-throughput sequencing data processing and analysis

Metagenomic data generated for the air samples were processed for adaptor removal and quality trimming with a Phred quality score threshold of Q20 using Cutadapt v. 1.8.1^30^. Two million reads (250 bp) were randomly selected from each sample as a representative set and aligned against the NCBI non-redundant (NR) protein database downloaded on 7/08/2017 using the alignment tool RAPSearch v. 2.15^21,22^.

Resulting alignments were imported into MEGAN v.5.11.3, which assigns taxon IDs based on the NCBI taxonomy^23,24^. To achieve the desired taxonomic specificity, we used the following filtering parameters: min score = 100 (bit score), max expected = 0.01 (e-value), top percent = 10 (top 10% of highest bit score), min support = 25 (minimum number of reads required for taxonomic assignment), LCA percent = 100 (naive), min complexity = 0.33 (sequence complexity). Lowest common ancestry (LCA) for each read on the NCBI taxonomy is assigned using MEGAN’s LCA algorithm. In instances where all of the above filtering criteria have been fulfilled, reads are assigned to levels of taxonomic classification ranging from domain to species. In our study, species-level classification is only reached if at least 25 reads uniquely align to a single species in the database with a 100% match on the protein level over at least 50% of the 250 bp read. Due to limits of existing public sequence databases, some sequencing reads did not result in meaningful alignments and were assigned to the ‘no-hits’ category. Unassigned reads are sequencing reads for which low-complexity, repetitive DNA sequences or multiple alignments beyond domain-level are encountered.

### Statistical analysis

Seasonal difference in richness, evenness and relative abundances of the microbial taxa was assessed by generalized regression modelling in R v. 3.3.3^31^. Multivariate linear modelling analysis was performed in *mvabund* package in R v. 3.3.3. To visualize multivariate patterns in microbial communities, Bray–Curtis dissimilarity distances among centroids for each sample series were calculated in *vegan* package in R v. 3.3.3. Principal Coordinates (PCo) were used as an ordination method. Alfa diversity indices *chao1* and Simpson E were calculated in QIIME v. 1.8.0^32^. Cross-correlation analysis was performed in R v.3.3.3.

### Meteorological data

The retrospective meteorological data of the local weather station were downloaded from the open source service “Weather and Climate” (http://www.pogodaiklimat.ru/, weather archive information downloaded on 2.10.2018). Meteorological characteristics (temperature, relative humidity, and wind direction) during the time series are represented in Fig. S1 and Fig. S2.

## Supplementary information

Supplementary Information.

## Data Availability

Raw metagenomic sequencing data have been submitted to NCBI (BioProject ID: PRJNA643946, study ID: SRP270871).

## References

[1] Gusareva ES (2019). Microbial communities in the tropical air ecosystem follow a precise diel cycle. Proc. Natl. Acad. Sci. USA.

[2] Franzetti A, Gandolfi I, Gaspari E, Ambrosini R, Bestetti G (2011). Seasonal variability of bacteria in fine and coarse urban air particulate matter. Appl. Microbiol. Biotechnol..

[3] Bowers RM (2013). Seasonal variability in bacterial and fungal diversity of the near-surface atmosphere. Environ. Sci. Technol..

[4] Fierer N (2008). Short-term temporal variability in airborne bacterial and fungal populations. Appl. Environ. Microbiol..

[5] Yooseph S (2013). A metagenomic framework for the study of airborne microbial communities. PLoS ONE.

[6] Radosevich JL, Wilson WJ, Shinn JH, DeSantis TZ, Andersen GL (2002). Development of a high-volume aerosol collection system for the identification of air-borne micro-organisms. Lett. Appl. Microbiol..

[7] Fahlgren C, Bratbak G, Sandaa RA, Thyrhaug R, Zweifel UL (2011). Diversity of airborne bacteria in samples collected using different devices for aerosol collection. Aerobiologia.

[8] Bowers RM (2011). Sources of bacteria in outdoor air across cities in the midwestern United States. Appl. Environ. Microbiol..

[9] Brodie EL (2007). Urban aerosols harbor diverse and dynamic bacterial populations. Proc. Natl. Acad. Sci. USA.

[10] Fiegel J, Clarke R, Edwards DA (2006). Airborne infectious disease and the suppression of pulmonary bioaerosols. Drug Discov. Today.

[11] Mac Aogain M (2019). Distinct “Immunoallertypes” of disease and high frequencies of sensitization in non-cystic fibrosis bronchiectasis. Am. J. Respir. Crit. Care Med..

[12] Tiew PY (2020). The mycobiome in health and disease: Emerging concepts, methodologies and challenges. Mycopathologia.

[13] Van Dyken SJ (2011). Fungal chitin from asthma-associated home environments induces eosinophilic lung infiltration. J. Immunol..

[14] Zukiewicz-Sobczak WA (2013). The role of fungi in allergic diseases. Postepy Dermatol. Alergol..

[15] Xu X (2012). Strain-dependent induction of neutrophil histamine production and cell death by *Pseudomonas aeruginosa*. J. Leukoc. Biol..

[16] Sache IA, de Vallavieille-Pope C (1995). Classification of airborne plant pathogens based on sporulation and infection characteristics. Can. J. Bot..

[17] Pandey R (2016). Ice-nucleating bacteria control the order and dynamics of interfacial water. Sci. Adv..

[18] Andreeva IS (2001). Variability of the content of live microorganisms in the atmospheric aerosol in southern regions of western Siberia. Dokl. Biol. Sci..

[19] Safatov AS (2010). Atmospheric aerosol fungi concentration and diversity in the South of Western Siberia. Atmos. Ocean. Opt..

[20] Jiang C (2018). Dynamic human environmental exposome revealed by longitudinal personal monitoring. Cell.

[21] Ye Y, Choi JH, Tang H (2011). RAPSearch: A fast protein similarity search tool for short reads. BMC Bioinform..

[22] Zhao Y, Tang H, Ye Y (2012). RAPSearch2: A fast and memory-efficient protein similarity search tool for next-generation sequencing data. Bioinformatics.

[23] Huson DH, Auch AF, Qi J, Schuster SC (2007). MEGAN analysis of metagenomic data. Genome Res..

[24] Huson DH (2016). MEGAN community edition—Interactive exploration and analysis of large-scale microbiome sequencing data. PLoS Comput. Biol..

[25] Pepys J (1963). Farmers lung thermophilic actinomycetes as a source of farmers lung hay antigen. Lancet.

[26] Mikhailovskaya IF (2017). Long-term measurements (2010–2014) of carbonaceous aerosol and carbon monoxide at the Zotino Tall Tower Observatory (ZOTTO) in central Siberia. Atmos. Chem. Phys..

[27] Andreeva IS (2001). Seasonal variations in the microorganisms concentration in the biogenic component of atmospheric aerosol in the South of Western Siberia. J. Aerosol Sci..

[28] Andreeva IS (2018). Spore forming bacteria isolated from atmospheric aerosols in southwestern Siberia during atmospheric transfer of air masses. Bull. Nizhnevartovsk State Univ..

[29] Luhung I (2015). Protocol improvements for low concentration DNA-based bioaerosol sampling and analysis. PLoS ONE.

[30] Martin M (2011). Cutadapt removes adapter sequences from high-throughput sequencing reads. EMBnet J..

[31] R Core Team (2017). R: A Language and Environment for Statistical Computing.

[32] Caporaso JG (2010). QIIME allows analysis of high-throughput community sequencing data. Nat. Methods.

