# Supplementary material for: Taxonomic composition and seasonal dynamics of the air microbiome in West Siberia
Source: Sci Rep. 2020 Dec 9;10:21515. doi: 10.1038/s41598-020-78604-8 (PMC7726148; doi:10.1038/s41598-020-78604-8)
Supplement: Supplementary file 1 — Supplementary Information. [file 41598_2020_78604_MOESM1_ESM.docx]

**SUPPLEMENTARY TABLES AND FIGURES**

**Taxonomic composition and seasonal dynamics of the air microbiome in West Siberia**

**Elena S. Gusareva^1^*, Nicolas P.E. Gaultier^1^, Balakrishnan N. V. Premkrishnan^1^, Carmon Kee^1^, Serene Boon Yuean Lim^1^, Cassie E. Heinle^1^, Rikky W. Purbojati^1^, Ang Poh Nee^1^, Sachin R. Lohar^1^, Koh Yanqing^1^, Vladimir N. Kharkov^2^, Daniela I. Drautz-Moses^1^, Vadim A. Stepanov^2^ and Stephan C. Schuster^1^***

^1^Singapore Centre for Environmental Life Sciences Engineering (SCELSE), Nanyang Technological University, 60 Nanyang Drive, 637551 Singapore; ^2^Institute of Medical Genetics, Tomsk National Medical Research Centre, Russian Academy of Sciences, Tomsk 634050, Russian Federation.

*email: [egusareva@ntu.edu.sg](mailto:egusareva@ntu.edu.sg) and [SCSchuster@ntu.edu.sg](mailto:SCSchuster@ntu.edu.sg)

**Samples in the time-series**

**Table S1. Distribution of air samples collected during time-series experiments.**

| **experiment** | **season** | **sunrise** | **day** | **sunset** | **night** | **total** |
| --- | --- | --- | --- | --- | --- | --- |
| SUMMER 2017 | summer | - | 8 | - | 4 | 12 |
| WINTER 2017 | winter | 8 | 8 | - | 6 | 22 |
| SUMMER 2018 | summer | - | 20 | 12 | 12 | 44 |

SUMMER 2017 and 2018, WINTER 2017 – time-series experiments in Yurga, Russia.

We extracted DNA from all 78 air samples collected for 39 time intervals. For the first 34 samples, the filters were extracted separately, but the resulting DNA was combined for the library construction. For the remaining 44 samples, sufficient DNA was obtained.

**Meteorological characteristics during summer and winter time-series.**


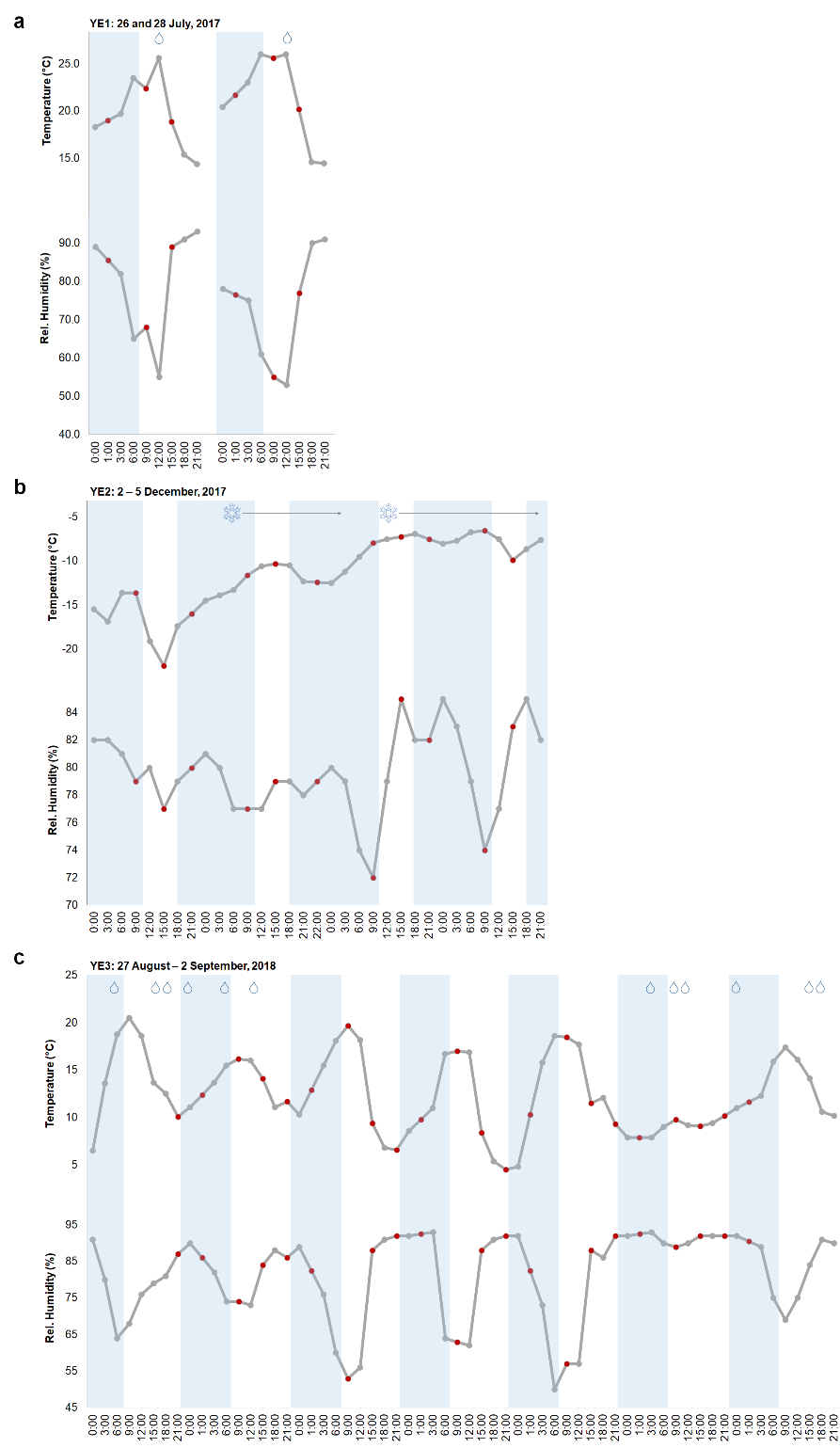
**Fig. S1 Meteorological characteristics during summer and winter time-series sampling.** Distributions of temperature and relative humidity (RH) during summer 2017 and 2018 (**a, b**), winter 2017 (**c**) time-series. Rain events and snowfall are indicated by the corresponding drop-shaped and snowflake-shaped symbols. Periods of night-time are indicated by a shaded background. The retrospective meteorological data of the local weather station were downloaded from the open source service “Weather and Climate” (<http://www.pogodaiklimat.ru/>). The red dots indicate beginning of the 2 h sampling intervals during the time-series experiments.

In summer, the warmest time of a day with the lowest relative humidity (RH) is after sunrise and lasts until noon when the temperature starts to drop and RH increases; the coldest time with the highest RH is between 21:00 and 1:00 in the morning.

In winter, dramatic turbulences in temperature and RH are frequently encountered. In the winter time-series during the first sampling day, the temperature dropped to -22^o^C with RH of 77%. Then, the weather changed to a warmer snowy period, with the temperature gradually increasing over three days to -7^o^C, while the RH oscillated in a wider range (72-85%) with the highest values at noon and lowest at about 9:00 in the morning.


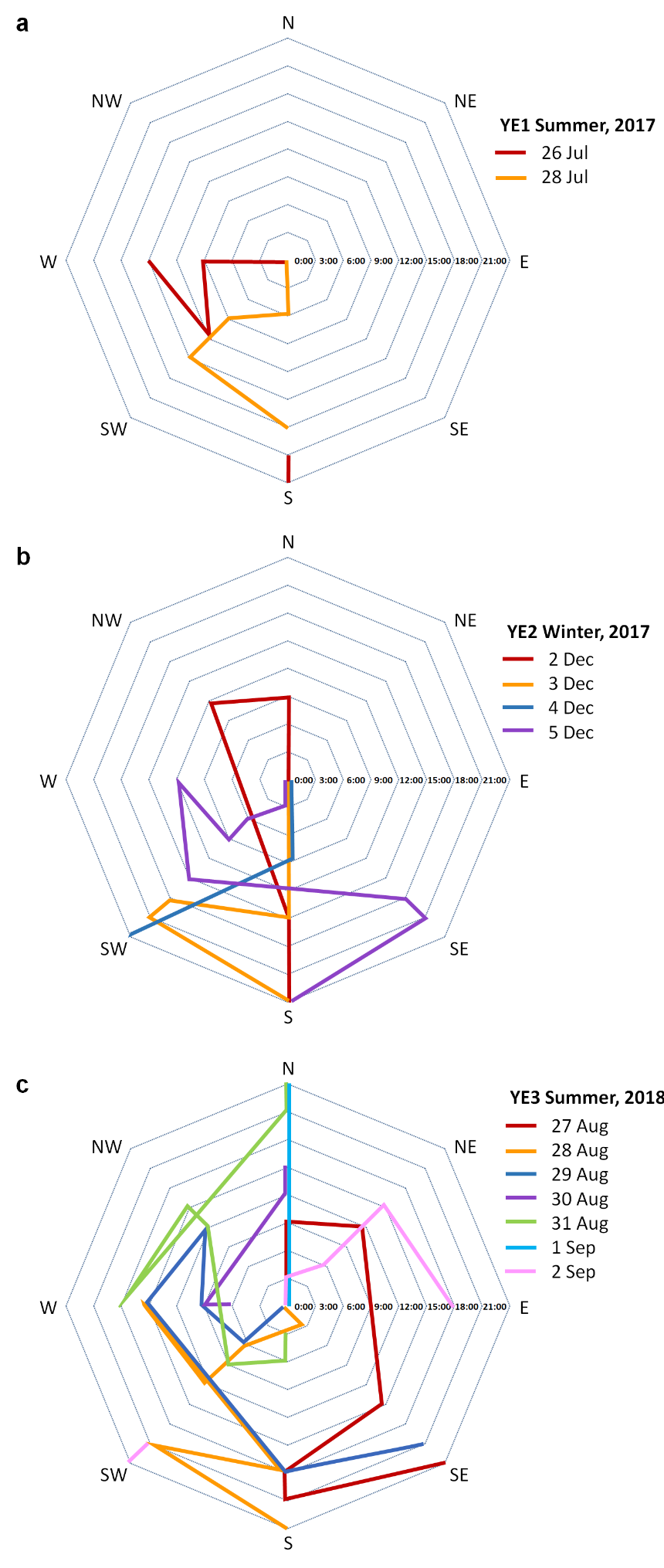
**Fig. S2 Trajectories of wind direction during summer and winter time-series experiments.**

Summer 2017 and 2018 (**a, b**), winter 2017 (**c**) time-series. Different colors of trajectories indicate different days of the experiments. Trajectories are interrupted during calm periods of a day. The retrospective meteorological data of the local weather station was downloaded from the open source service “Weather and Climate” (<http://www.pogodaiklimat.ru/>).

The retrospective analysis of the wind trajectories shows that air often flows from different directions and tends to form a vortex in this region. The only day in the course of the experiments when the wind was blowing in a single direction from north was on September 1, 2018. On this day, the atmospheric temperature was cold and did not exceed 10.2 ^o^C.

**Seasonal and diel dynamics of the airborne communities**

**Fig. S3 Abundance and taxonomic structure of the microbial communities.**

**
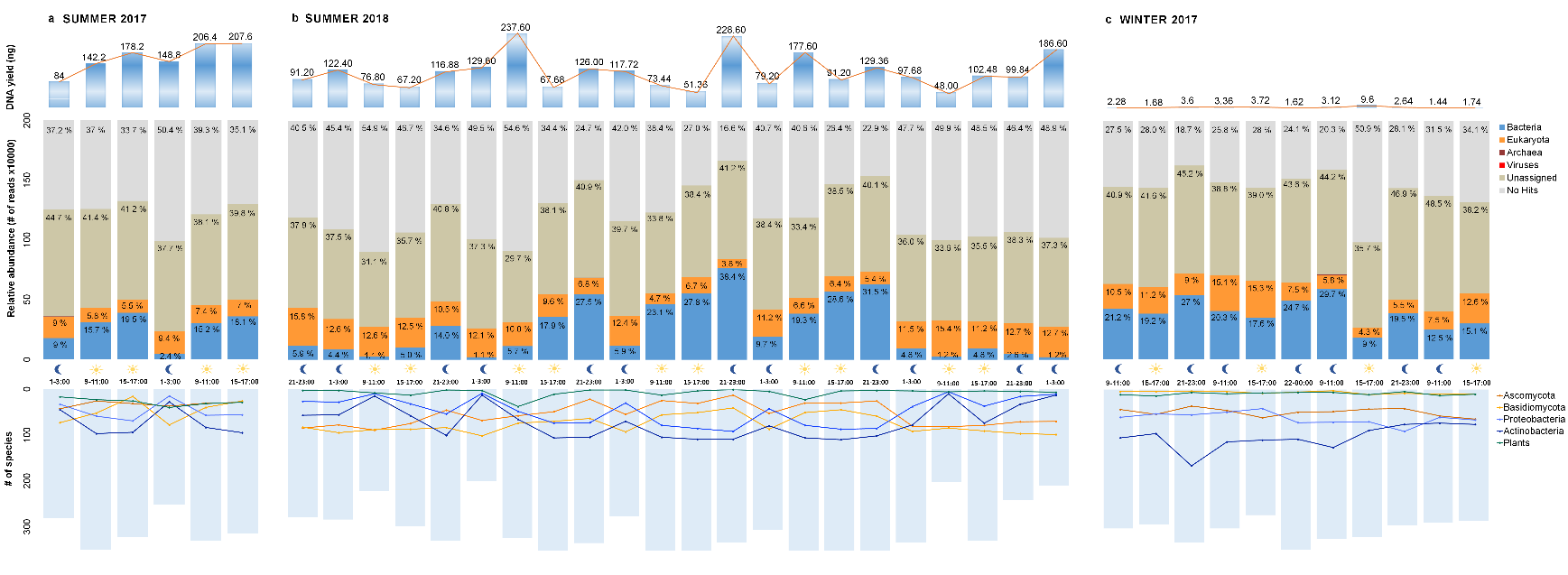
**

The biomass was collected with two SASS3100 filter-based air samplers at 300 L/min air flowrate for 2 h. Sampling time periods are shown; night and day samples are indicated with corresponding symbols next to the sample names. Upper panels: the total collected DNA yields in ng per time-point. Middle panels: relative abundances of microbes at the super-kingdom level taxonomical classification represented by the number of assigned reads (colored bars) and by percentages. Lower panel: richness of the microbial community represented by the total number of identified species (light blue bars); richness of bacteria (Proteobacteria and Actinobacteria) and eukaryotes (plants and fungi Ascomycota and Basidiomycota) represented by the colored lines.

**Fig. S4 Intersection between summer (green) and winter (blue) time-series sampling for bacterial species (a) and fungi (b).** Numbers of assigned species are indicated.

**
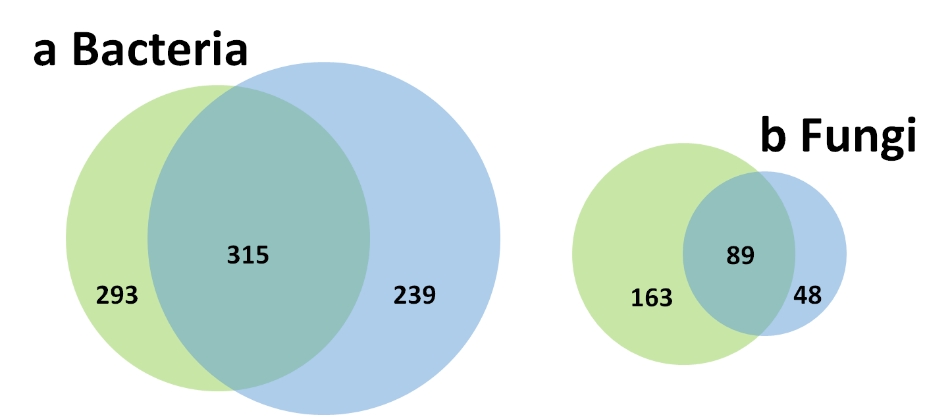
**

**Table S2. Categories of the top-50 microbial species identified in the air in summer and in winter.**

| **Category** | **Species** | **Taxa** | **AVG Rel. Abundance (%)** | |
| --- | --- | --- | --- | --- |
|  |  |  | **SUMMER** | **WINTER** |
| **human (opportunistic) pathogens and allergens** | *Alternaria alternata* | Ascomycota | 0.09 | 0.16 |
|  | *Aspergillus cristatus* | Ascomycota | 0 | 0.59 |
|  | *Aspergillus fumigatus* | Ascomycota | 0 | 0.05 |
|  | *Aspergillus nidulans* | Ascomycota | 0 | 0.21 |
|  | *Aspergillus ruber* | Ascomycota | 0 | 0.2 |
|  | *Aureobasidium pullulans* | Ascomycota | 0.02 | 0.18 |
|  | *Botrytis cinerea* | Ascomycota | 0.2 | 0.03 |
|  | *Corynebacterium xerosis* | Actinobacteria | 0 | 0.02 |
|  | *Massilia timonae* | Proteobacteria | 0 | 0.02 |
|  | *Plasmodium ovale* | Protozoa | 0 | 0.02 |
|  | *Saccharopolyspora rectivirgula* | Actinobacteria | 0 | 0.32 |
|  | *Sphingomonas sp. URHD0057* | Proteobacteria | 0.03 | 0.01 |
|  | *Trichinella patagoniensis* | Nematoda | 0 | 0.02 |
|  | *Verruconis gallopava* | Ascomycota | 0.01 | 0 |
|  | *Wallemia mellicola* | Basidiomycota | 0 | 0.17 |
| **plant pathogens** | *Alternaria alternata* | Ascomycota | 0.09 | 0.16 |
|  | *Ascochyta rabiei* | Ascomycota | 0.07 | 0.01 |
|  | *Botrytis cinerea* | Ascomycota | 0.2 | 0.03 |
|  | *Ceraceosorus bombacis* | Basidiomycota | 0.02 | 0 |
|  | *Epicoccum nigrum* | Ascomycota | 0.09 | 0.07 |
|  | *Melampsora larici-populina* | Basidiomycota | 0.03 | 0.01 |
|  | *Microdochium bolleyi* | Ascomycota | 0.02 | 0 |
|  | *Parastagonospora nodorum* | Ascomycota | 0.1 | 0.02 |
|  | *Puccinia striiformis* | Basidiomycota | 0.05 | 0.02 |
|  | *Rhizoctonia solani* | Basidiomycota | 0.09 | 0 |
|  | *Stagonospora sp. SRC1lsM3a* | Ascomycota | 0.02 | 0.01 |
|  | *Stereum hirsutum* | Basidiomycota | 0.04 | 0 |
|  | *Ustilago hordei* | Basidiomycota | 0 | 0.03 |
| **plant endophytes** | *Aureobasidium pullulans* | Ascomycota | 0.02 | 0.18 |
|  | *Marmoricola sp. Leaf446* | Actinobacteria | 0.02 | 0.02 |
|  | *Marmoricola sp. URHB0036* | Actinobacteria | 0.01 | 0.01 |
|  | *Phialocephala scopiformis* | Ascomycota | 0.02 | 0.02 |
|  | *Rhizobacter sp. Root404* | Proteobacteria | 0.02 | 0.01 |
|  | *Sphingomonas sp. URHD0057* | Proteobacteria | 0.03 | 0.01 |
|  | *Sphingomonas sp. Leaf20* | Proteobacteria | 0 | 0.02 |
| **plants** | *Aegilops tauschii* | Streptophyta | 0.02 | 0.08 |
|  | *Daucus carota* | Streptophyta | 0.01 | 0 |
|  | *Helianthus annuus* | Streptophyta | 0.03 | 0.02 |
|  | *Hordeum vulgare* | Streptophyta | 0 | 0.02 |
|  | *Morus notabilis* | Streptophyta | 0.02 | 0 |
|  | *Oryza sativa* | Streptophyta | 0.01 | 0.02 |
|  | *Triticum urartu* | Streptophyta | 0 | 0.03 |
|  | *Vitis vinifera* | Streptophyta | 0.02 | 0 |
| **cold-related and stress environment microbes** | *Arthrobacter sp. L77* | Actinobacteria | 0.02 | 0.04 |
|  | *Cnuella takakiae* | Bacteroidetes | 0.03 | 0.01 |
|  | *Hymenobacter norwichensis* | Bacteroidetes | 0 | 0.02 |
|  | *Hymenobacter roseosalivarius* | Bacteroidetes | 0.01 | 0.02 |
|  | *Hymenobacter sp. PAMC 26554* | Bacteroidetes | 0.01 | 0.06 |
|  | *Kocuria polaris* | Actinobacteria | 0.02 | 0.04 |
|  | *Kocuria sp. UCD-OTCP* | Actinobacteria | 0.01 | 0.01 |
|  | *Rachicladosporium antarcticum* | Ascomycota | 0.08 | 0.06 |
|  | *Thermoactinomyces sp. CDF* | Firmicutes | 0 | 0.02 |
|  | *Thermobifida fusca* | Actinobacteria | 0 | 0.02 |
|  | *Wallemia mellicola* | Basidiomycota | 0 | 0.17 |
| **wood-rotting saprotrophs** | *Cylindrobasidium torrendii* | Basidiomycota | 0.02 | 0 |
|  | *Dichomitus squalens* | Basidiomycota | 0.05 | 0 |
|  | *Exidia glandulosa* | Basidiomycota | 0.02 | 0 |
|  | *Fomitopsis pinicola* | Basidiomycota | 0.03 | 0 |
|  | *Mycena chlorophos* | Basidiomycota | 0.02 | 0 |
|  | *Peniophora sp. CONT* | Basidiomycota | 0.08 | 0 |
|  | *Phanerochaete carnosa* | Basidiomycota | 0.03 | 0 |
|  | *Phlebia centrifuga* | Basidiomycota | 0.03 | 0 |
|  | *Phlebiopsis gigantea* | Basidiomycota | 0.03 | 0 |
|  | *Piloderma croceum* | Basidiomycota | 0.02 | 0 |
|  | *Postia placenta* | Basidiomycota | 0.02 | 0 |
|  | *Schizopora paradoxa* | Basidiomycota | 0.07 | 0 |
|  | *Stereum hirsutum* | Basidiomycota | 0.04 | 0 |
|  | *Tilletiaria anomala* | Basidiomycota | 0.01 | 0 |
|  | *Trametes pubescens* | Basidiomycota | 0.05 | 0 |
|  | *Trametes versicolor* | Basidiomycota | 0.03 | 0 |
|  | *Tulasnella calospora* | Basidiomycota | 0.01 | 0 |
| **mushrooms** | *Agaricus bisporus* | Basidiomycota | 0.02 | 0 |
|  | *Coprinopsis cinerea* | Basidiomycota | 0.02 | 0 |
|  | *Cordyceps militaris* | Basidiomycota | 0.02 | 0 |
|  | *Galerina marginata* | Basidiomycota | 0.02 | 0 |
|  | *Grifola frondosa* | Basidiomycota | 0.02 | 0 |
|  | *Hypholoma sublateritium* | Basidiomycota | 0.04 | 0 |
|  | *Hypsizygus marmoreus* | Basidiomycota | 0.06 | 0 |
|  | *Pleurotus ostreatus* | Basidiomycota | 0.06 | 0 |
| **airborne bacteria** | *Knoellia aerolata* | Actinobacteria | 0.05 | 0.02 |
|  | *Skermanella aerolata* | Proteobacteria | 0.01 | 0.01 |
|  | *Roseomonas aerilata* | Proteobacteria | 0.01 | 0.02 |
| **water-associated bacteria** | *Altererythrobacter troitsensis* | Proteobacteria | 0.02 | 0 |
|  | *Arsenicicoccus bolidensis* | Actinobacteria | 0.01 | 0.01 |
|  | *Microlunatus phosphovorus* | Actinobacteria | 0.04 | 0.03 |
|  | *Nakamurella multipartita* | Actinobacteria | 0.01 | 0.01 |
|  | *Paracoccus chinensis* | Proteobacteria | 0.02 | 0.01 |
|  | *Paracoccus sediminis* | Proteobacteria | 0.07 | 0.02 |
|  | *Sphingomonas astaxanthinifaciens* | Proteobacteria | 0.11 | 0.04 |
|  | *Sphingomonas jaspsi* | Proteobacteria | 0.02 | 0.01 |
| **soil bacteria** | *Bradyrhizobium sp. DFCI-1* | Proteobacteria | 0 | 0.03 |
|  | *Rubellimicrobium mesophilum* | Proteobacteria | 0.04 | 0.02 |
|  | *Promicromonospora sukumoe* | Actinobacteria | 0 | 0.01 |
|  | *Saccharopolyspora antimicrobica* | Actinobacteria | 0 | 0.03 |

**Fig. S5 Bubble chart of the top 50 airborne microbial species identified in summer 2017 (26 and 28 July 2017).**

The data are square-root transformed for this representation.

**
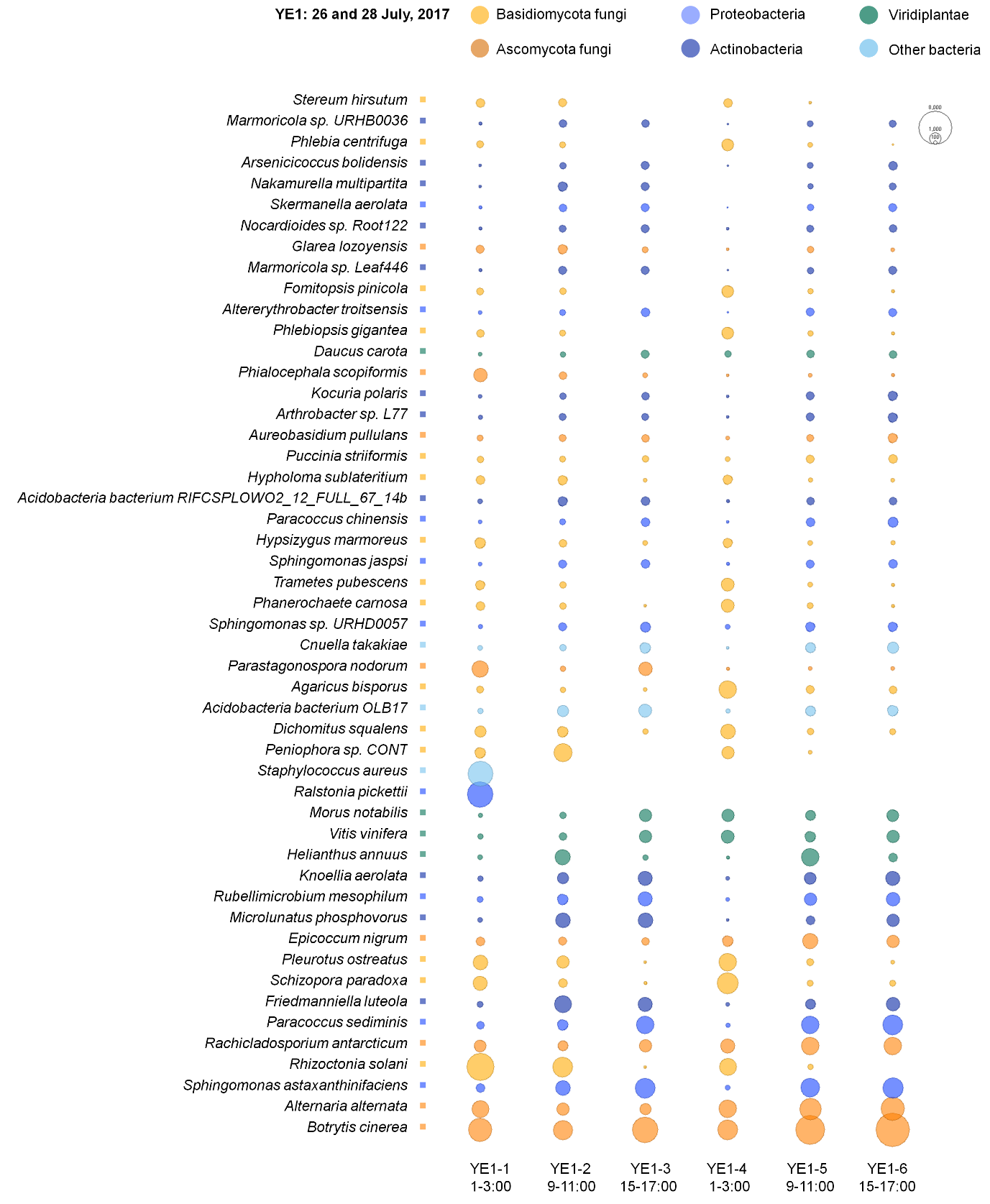
**

In the list of the most abundant organisms of the summer 2017 time series (first sample), we detected two opportunistic human pathogens, *Staphylococcus aureus* and *Ralstonia pickettii*, that cause bacterial infections in immunocompromised individuals. These microbes are also commensal bacteria usually colonizing mucosal and skin surfaces, and are therefore likely to be contamination from experimental handling.

**Fig. S6 Bubble chart of the top 50 airborne microbial species identified in summer 2018 (27 August – 2 September 2018).**

Read counts for pairs of technical replicates collected at the same time intervals and location, but processed and sequenced separately. The data are square-root transformed for this representation.

**
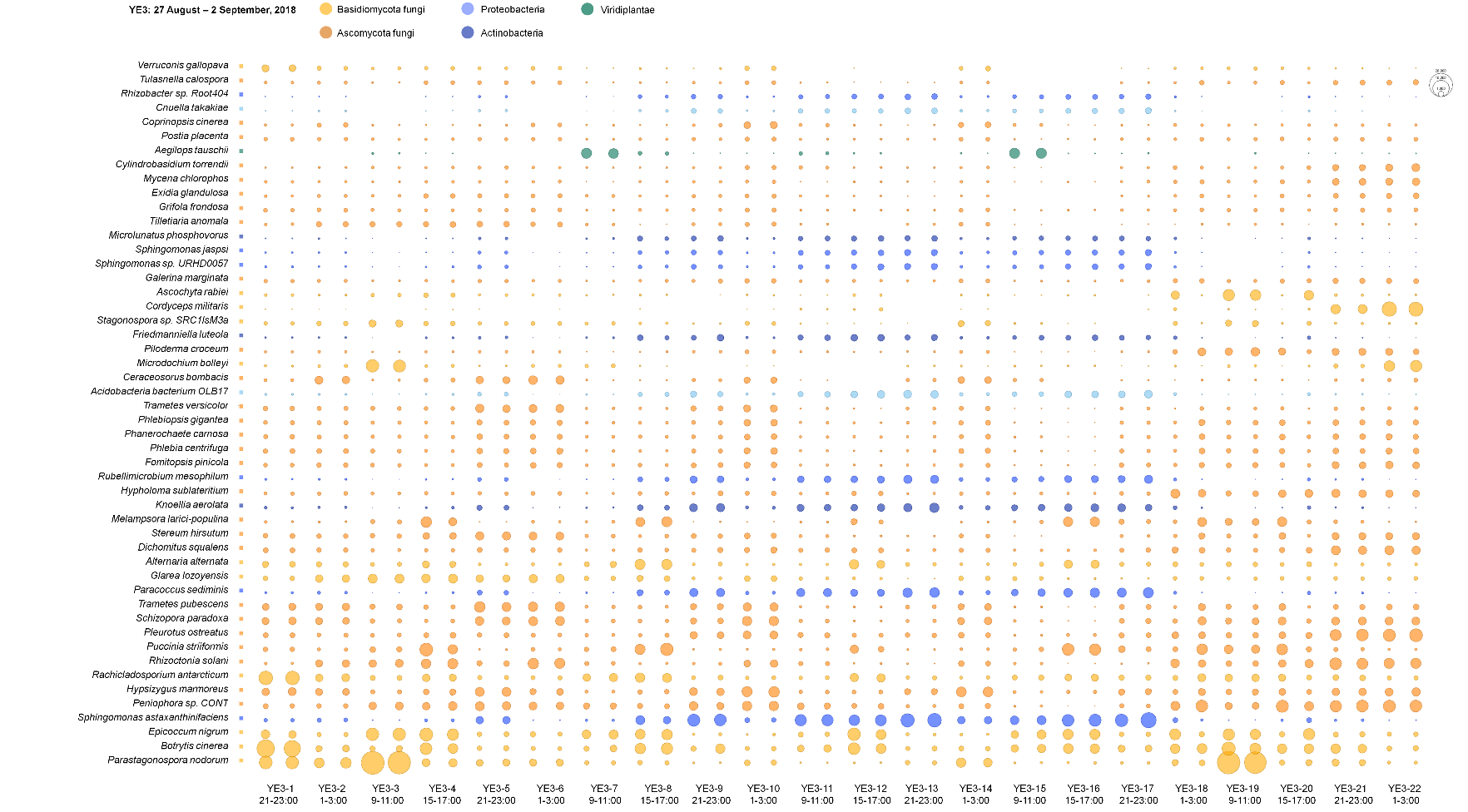
**

**Fig. S7 Diel variation of the airborne microbes in summer.**

Significant diel changes of the relative abundances of some airborne microbial taxa (*p*_Multivariate_GLM_-value < 0.05) in summer 2018.

**
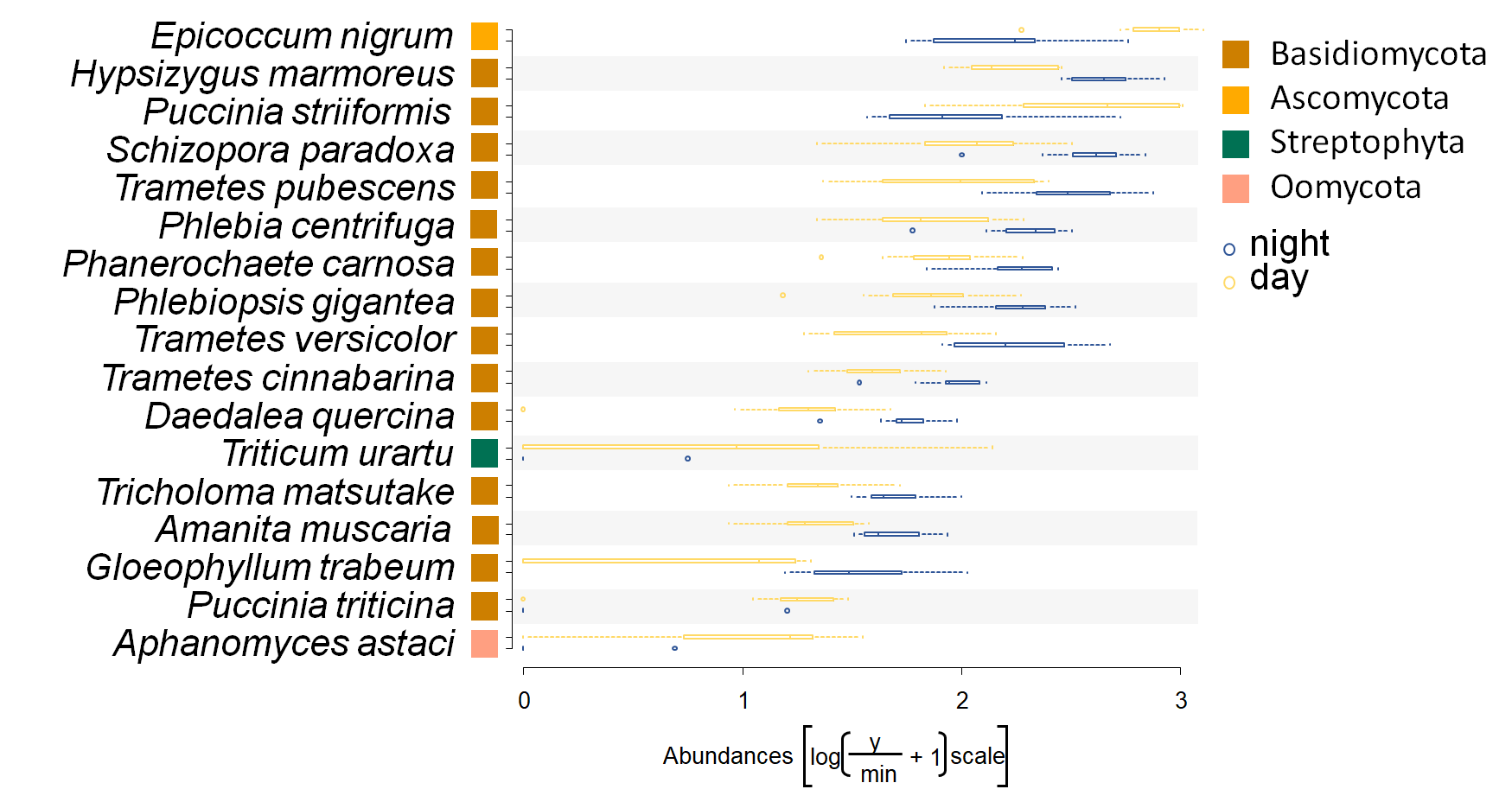
**

**
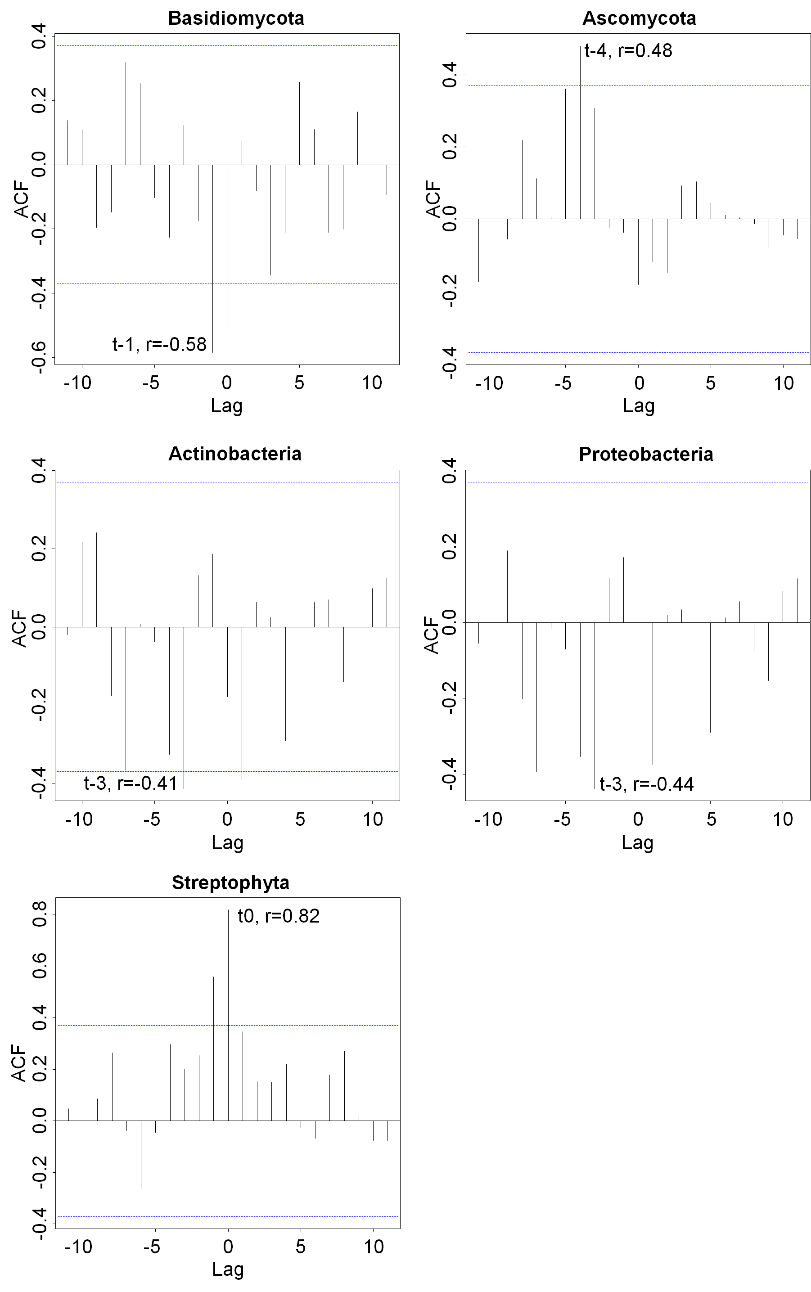
**

**Fig. S8 Cross-correlation function (CCF) analysis of the atmospheric temperature profiles with five taxa**

This analysis allows for the determination of the lag at which the correlation between two time series is strongest.

The cross-correlation function plots yield correlation values (r) for different lag orders (t) that can go in either direction, being positive or negative.

The dashed blue line indicates the level of statistical significance for the correlation.

The strongest cross-correlation was identified for Basidiomycota fungi (*r* = -0.58 at t-1) and Streptophyta plants (*r* = 0.82 at t0).

**Fig. S9 Bubble chart of the top 50 airborne microbial species identified in winter 2017 (2 – 5 December 2017).**

The data are square-root transformed for the representation.

**
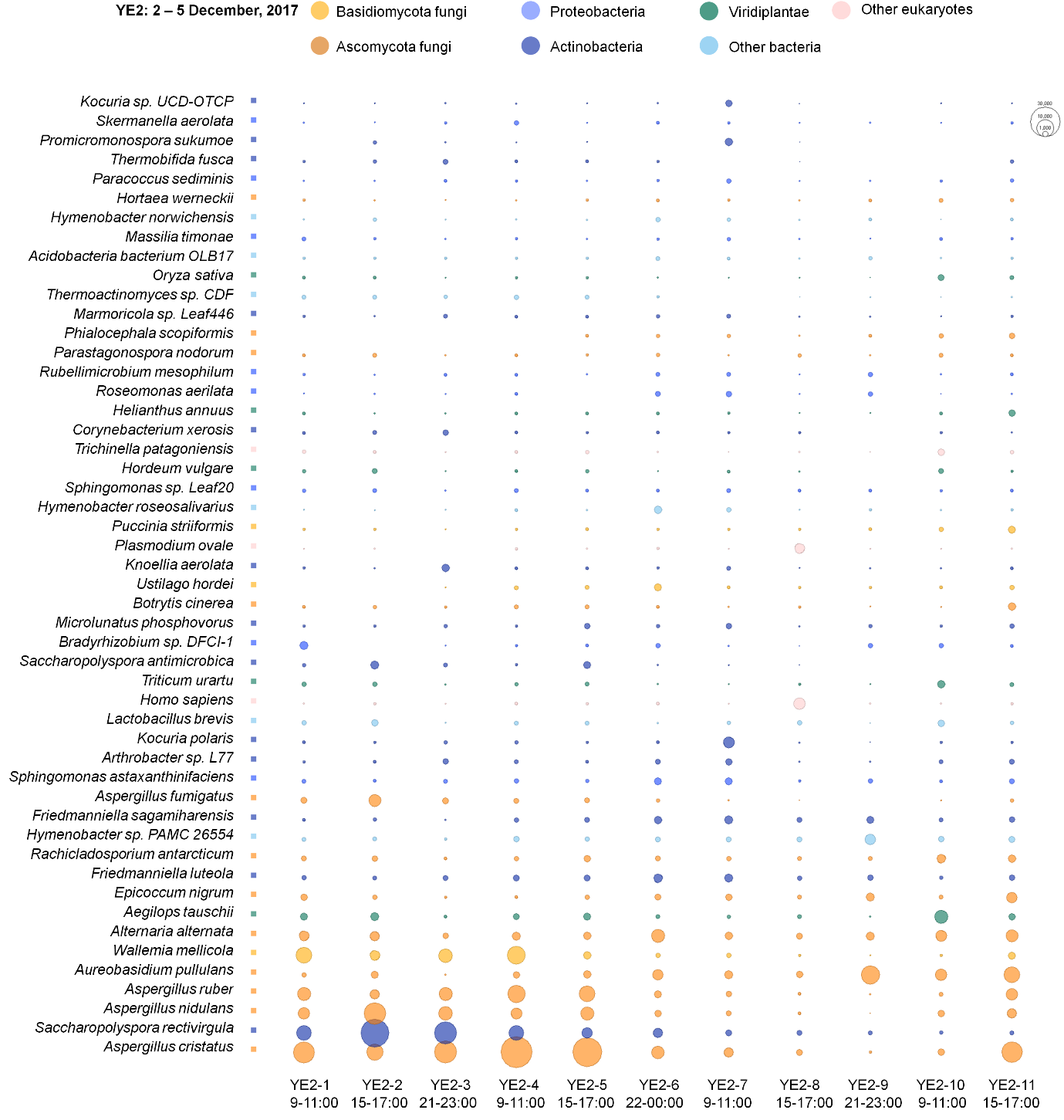
**

**Fig. S10 Richness and evenness of the airborne communities in summer and winter.**

**
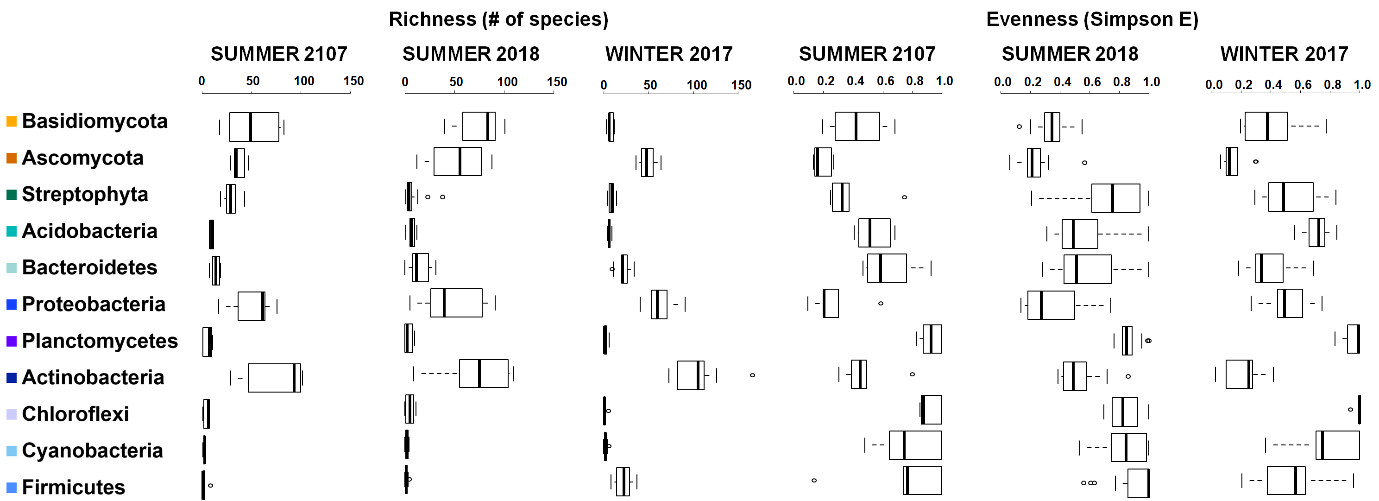
**

**Table S3 Seasonal difference in richness and evenness of the microbial taxa.**

Results for association analysis assessed by regression modeling.

| **Taxa** | ***p*_rm_-values** | | |
| --- | --- | --- | --- |
|  | **Richness** | **Evenness** | **Relative abundance** |
| Basidiomycota | 6.9*10^-11^ | 0.625 | 6.4*10^-6^ |
| Ascomycota | 0.84 | 0.056 | 0.0003 |
| Sreptophyta | 0.46 | 0.151 | 0.433 |
| Acidobacteria | 0.15 | 0.003 | 0.385 |
| Bacterioidetes | 0.02 | 0.003 | 0.139 |
| Proteobacteria | 0.11 | 0.006 | 0.143 |
| Planctomycetes | 0.11 | 0.03 | 0.848 |
| Actinobacteria | 0.01 | 1.8*10^-7^ | 0.028 |
| Chloroflexi | 0.003 | 0.0008 | 0.124 |
| Cyanobacteria | 0.78 | 0.71 | 0.009 |
| Firmicutis | 7.3*10^-13^ | 8.8*10^-5^ | 3.5*10^-13^ |

**Analysis of blanks**

**Fig. S11 Bubble chart of the top 50 concomitant microbial taxa identified in blanks across all time-series experiments.**


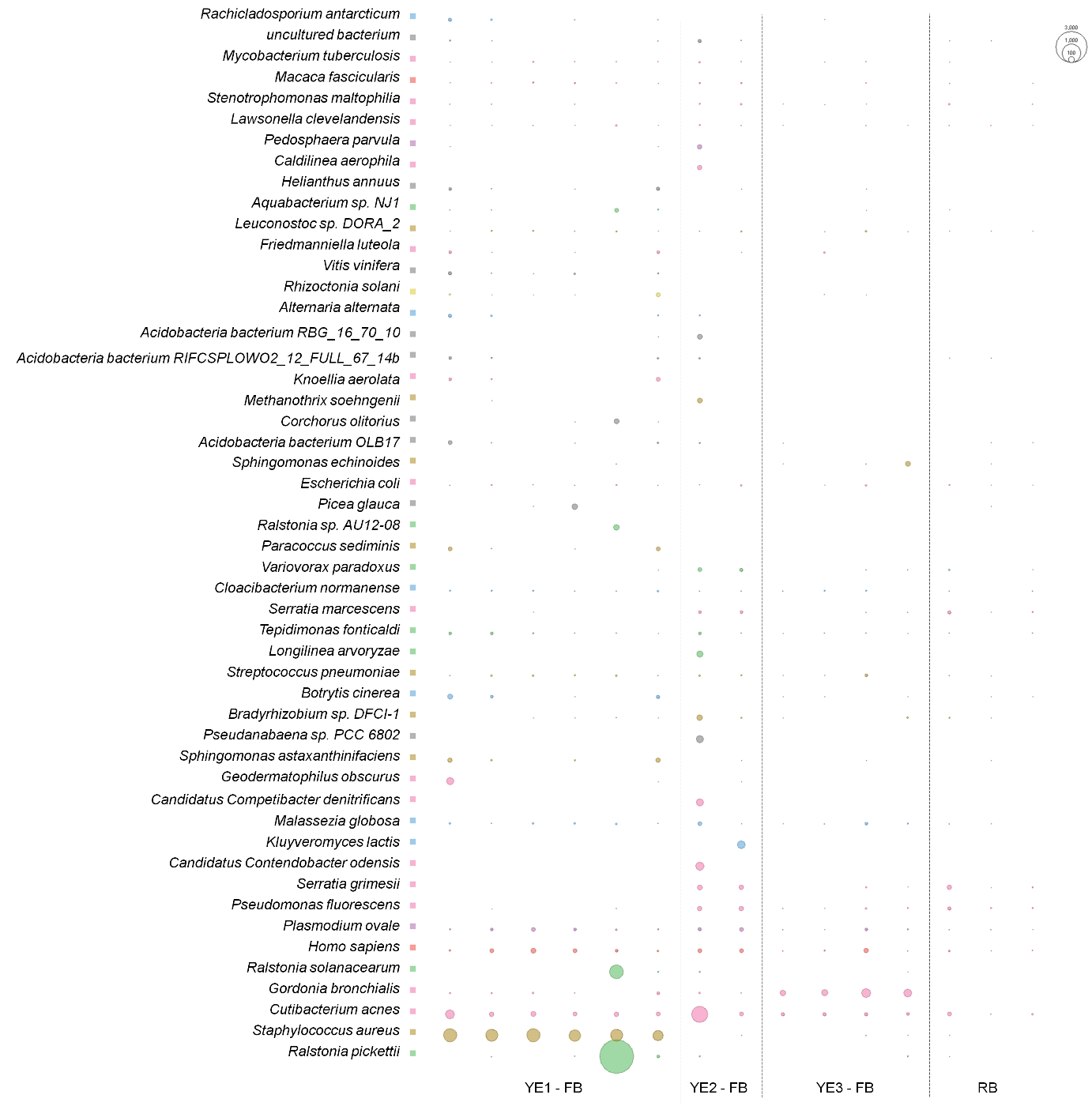


In total, 12 filter blank samples (FB) and three reagent blanks (RB) were collected and analyzed. Generally, blanks pick up trace amounts of biomass. Particularly, the total DNA yields of the blank samples were up to 0.032 ng/µl, which is at least ~6-fold lower than the least concentrated time-series sample. For next-generation sequencing library preparation, the PCR cycles for the blanks were normalized to eight.

The concomitant taxa that most frequently appear in blanks were human commensal bacteria colonizing mucosal and skin surfaces, particularly *Staphylococcus aureus, Cutibacterium acnes,* and *Ralstonia pickettii*. Human assigned reads were also found.
